# Color stability of Bulk-Fill composite restorations

**DOI:** 10.4317/jced.57579

**Published:** 2020-11-01

**Authors:** Marianna-Falcão Silva, Marlon-Ferreira Dias, Paulo-Cardoso Lins-Filho, Claudio-Heliomar-Vicente Silva, Renata-Pedrosa Guimarães

**Affiliations:** 1 Graduated in Dentistry from Federal University of Pernambuco; 2 Master student of the Dentistry postgraduate program of Universidade Estadual Paulista; 3 PhD student of the Dentistry postgraduate program of Federal University of Pernambuco; 4 Associate Professor at Federal University of Pernambuco

## Abstract

**Background:**

The color stability of the composite resin is an important property that influences its clinical longevity, which remains an inherent challenge to the material. Thus, the purpose of this study was to evaluate the color stability of bulk-fill resins when exposed to dye.

**Material and Methods:**

Cavities were prepared in 80 bovine incisors, which were randomly assigned into 4 groups (n = 20) according with the resin composite used: P60 (Control Group - Filtek P60, 3M/ESPE), FP (Filtek Bulk-Fill Posterior, 3M/ESPE), SDR (SDR, Dentsply) and FF (Filtek Bulk Fill Flow, 3M/ESPE). All restorations were performed according to the protocol of each manufacturer, the control group was restored using the incremental technique, and the other groups using single-increment technique. The color of each restoration was measured using a portable digital spectrophotometer (Easyshade-Vita) according to the CIELab system, and then the teeth were submerged in red wine for 07 days, kept in a biological oven at 37ºC. New color registration was performed to measure the ΔE index of color variation.

**Results:**

The P60 group had the lowest average ΔE (16.96), while the FF group had the highest average (28.09) and ranged from 21.19 to 26.28 in the FP and SDR groups.

**Conclusions:**

Analysis of the color variation showed that the control group had better color stability than the Bulk-Fill resins evaluated.

** Key words:**Dental restoration failure, Food coloring agents, polymerization.

## Introduction

Composite resins have their indication for posterior teeth restoration established in clinical studies that found excellent performance (1). However, one of the main limitations of composite resins is related to the volumetric shrinkage resulting from polymerization (2), a property inherent in polymeric materials. This property results in stress forces at the tooth-restoration interface, a consequence generally reduced using a specific cavity insertion protocol known as the incremental insertion technique (1,3). When these polymerization shrink forces are greater than adhesion forces, cracks may be generated and the risk of caries recurrence and failure increases (4).

The incremental insertion technique requires a longer working time, especially in deep cavities, since the maximum incremental thickness has been 2mm. In addition, it is a relatively sensitive technique, whit an increased risk of contamination by mouth fluids and air bubble formation between increments (5). Furthermore, bond failure between composite layers and difficulty in insertion into small cavities may also occur (6,7).

Modifications in the formulation of resin composites have been made to overcome these deficiencies, facilitate the procedure, and increase the longevity of dental restorations. Among these new formulations, the most recent evolution that was the release of the so-called Bulk-Fill composites or low shrinkage resins or single increment resins with the proposal to fill 4-5mm cavities at once (5), without influencing the polymerization contraction, the degree of conversion or cavity adaptation (8).

In general, the main property that characterizes this material is the low degree of shrinkage after polymerization (7) as well as the ability to compensate for the high C-factor of some cavities in posterior teeth, resulting in a significant reduction of clinical working time (1,8,9).

While manufacturers recommend a single 4mm increment fill, many clinicians suspect that the cure depth and mechanical properties may not be satisfactory. The color stability of these resins, for example, is a concern (10,11). Although there are several studies on the effect of different beverages on the color stability of composite resins, there is a lack of evidence on the color stability of Bulk-Fill resins that have been introduced for thicker layer application (12).

Low polymer migration can predispose to the phenomenon of fluid sorption and, consequently, the loss of stability over time. Once these new resins prove the quality of their properties, they will represent a great advance for adhesive restorations since they allow a restorative technique with great simplicity in a reduced chair time (1,8).

Thus, this study aimed to evaluate the color stability of different low shrinkage resins when in contact with a dye substance. Seeking a better comprehension of the polymers conversion efficiency when using the single-increment technique and if the type of monomer of these resins interferes with their chemical stability and thus increasing the absorption of food dyes.

## Material and Methods

This study was submitted and approved by the Animal Research Ethics Committee (protocol n. 23076.016531/2016).

For this experimental study, 80 bovine incisors with coronary integrity were selected, the absence of wear facets or pathological alterations of enamel was observed in stereoscopic magnifying glass. The teeth were submerged in a 0.5% chloramine solution for 7 days for disinfection, and their extrinsic stains and organic deposits removed by curette scraping, pumice paste prophylaxis. The specimens were stored in 0.9% physiological solution under refrigeration for no more than six months.

The teeth were sectioned 1mm beyond the cementoenamel junction and had the pulp remains removed and the root canal entrance sealed with composite. The specimens were then randomly distributed into 4 groups (n=20) according to the restorative material used. The characteristics of each composite are presented on  Table 1.

**Table 1:**
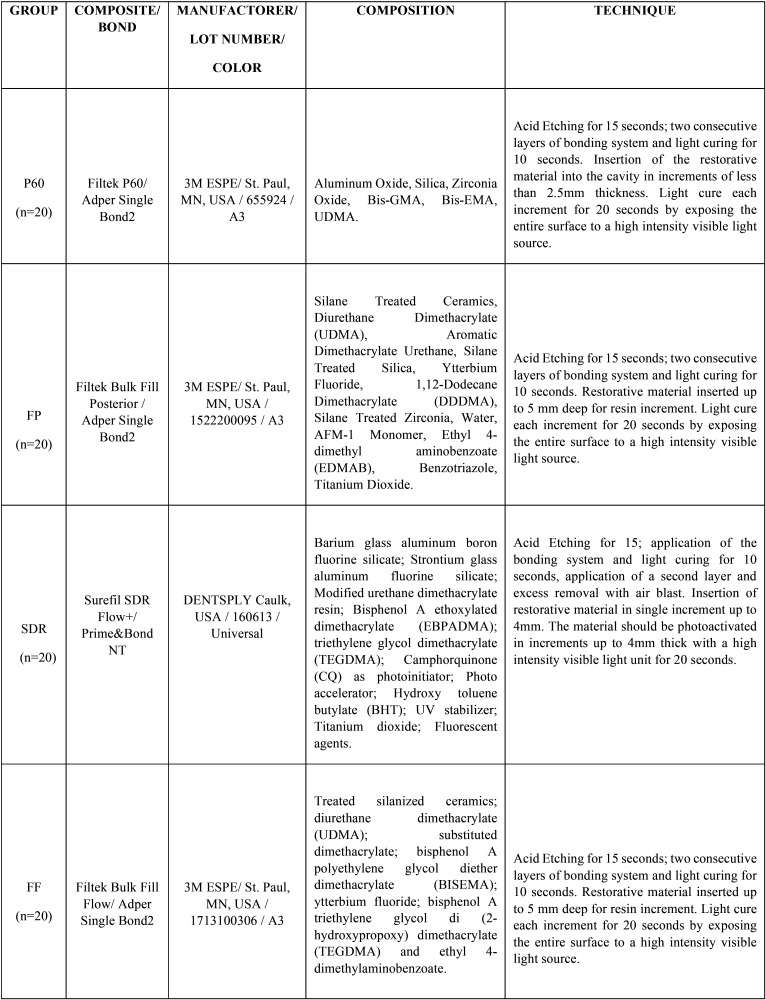
Protocol of the studied resin composites.

Cavity preparation was performed with a cylindrical diamond drill #3145 (KG Sorensen) under refrigeration, in the middle third of the buccal faces of each tooth measuring 4mm mesio-distal width X 3mm cervical-incisal height X 4mm deep, which was measured with the aid of a periodontal probe. The dimensions of the preparation were standardized through an acetate matrix cast, placed over the teeth, and marked with a pencil.

After prophylaxis with pumice paste and water, the cavities were dried with cotton pellets and conditioned with 37% phosphoric acid (Condac-FGM) for 15seconds, washed with water/air spray for 15seconds and dried again with slightly moistened cotton pellets. For each group, the bonding system recommended by the manufacturer was applied, which was applied in two consecutive layers followed by photoactivation during the time recommended by each manufacturer with high power LED device (Radii Cal / SDI).

The restorations were executed according to the protocol of each composite resin (Table 1), the control group was restored by the incremental technique and the others with the single increment technique. The teeth were stored in physiological solution for 24 hours and then the thickness of each tooth was measured using a digital thickness gauge. Then the finishing and polishing step was performed with low abrasive discs for composites (Praxis/TDV).

The color of each restoration was measured with a portable digital spectrophotometer. (Easyshade-Vita) according to CIELab system. Before measuring samples’ colors, the Easyshade Vita colorimeter was calibrated according to the manufacturer’s instructions. The probe tip was placed perpendicular and well-adjusted to the specimens’ surface to make accurate measurements. To standardize the color measurement site, a 2mm Ethylene / Vinyl Acetate copolymer matrix was customized over the teeth (Whiteness – FGM). The matrix was perforated in the middle third of the tooth buccal surface.

After the first measurement, the teeth were waterproofed with the application of colorless nail polish on the buccal and lingual surface of each tooth. After 24 hours, the sample was submerged into red wine (pH approx. 3,0) for seven days and kept in a 37°C biological hothouse. Subsequently, a second color measurement was performed and its variation was measured by calculating ΔE using the formula: ΔE* = [ΔL*2 + Δa*2 + Δb*2 ]1/2, where ΔL*= L0-L1; Δa= a0-a1; Δb=b0-b1.

The data were submitted to statistical analysis, all tests were applied considering an error of 5% and the confidence interval of 95%, and the analyzes were carried out using SPSS software version 23.0 (SPSS Inc. Chicago, IL, USA). The data were analyzed descriptively and inferentially. The hypothesis of equality of variance was verified by the Shapiro-Wilk and the Levene test. For between-groups comparison, the F-test (ANOVA) was chosen when the hypothesis of normality of data was accepted, and the Kruskal-Wallis test in case of normality rejection. Tukey comparisons occurred when equality of variances between groups was verified.

## Results

The mean color variation (ΔE) was lower in P60 group (16.96), highest in the FF group (28.09) and ranged from 21.19 to 26.28 in FP and SDR groups, differences that, except for SDR and FF, were statistically significant. The ΔE results for the groups are shown in Table 2.

**Table 2:**
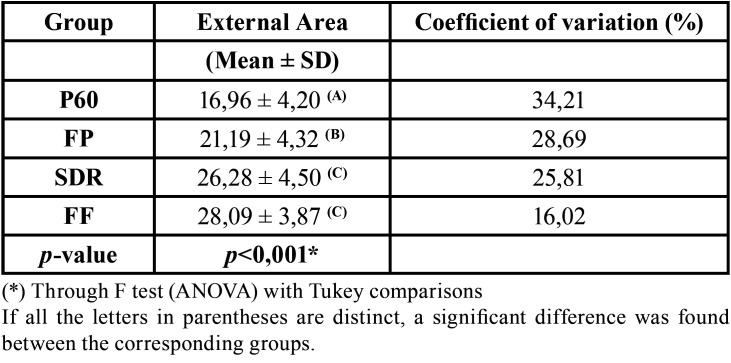
Mean and standard deviation of ΔE according to group and surface area.

## Discussion

With the increased demand for faster clinical procedures, Bulk-Fill composites allow working time reduction by decreasing the number of increments inserted into the cavity, with effective polymerization of layers up to 4 mm thick. This feature can be a great advantage in non-cooperative patients (1,8). Polymerization depth is an important parameter for Bulk-Fill resins evaluation. Studies attribute variations in the depth of polymerization to differences in color between different resins, as well as intensity and time of photoactivation (6,13).

The color stability of dental restorations is a parameter that can characterize resin materials in terms of longevity. In addition, compromised color stability may represent, indirectly, a low polymer conversion that depends on photoactivation process 13 and may result in material degradation and compromise restoration’s longevity (1,12).

The choice for red wine in this study was due to its high staining potential and low pH (3,0). Wine, as well as coffee and cola drinks are common beverages with high potential for staining restorative materials (10). Furthermore, it is known that low pH is a factor that negatively influences the physical and mechanical properties of conventional and Bulk-Fill composites (3).

The CIELab color system was chosen as dependent variable because it is a standard method for measuring color differences based on human perception. The ΔEvalue shows the relative color differences of dental materials or dental surfaces before and after an intervention. According to the literature, the values of ΔE <1 are considered not appreciable by the human eye. Values between 1 and 3.3 are considered clinically acceptable while ΔE values> 3.3 are not considered clinically accepTable (12). For all evaluated groups, ΔE obtained values greater than 10.0 proving the staining action of red wine on restorations.

The color variation analysis showed better stability for the control group (P60) compared to the Bulk-Fill resins tested (Table 2). This finding may correlate to a greater polymer conversion on P60, since in this resin there is UDMA, a monomer that increases the degree of polymer conversion (13). In contrast to this, lower polymerization shrinkage can facilitate dye penetration by cracking the tooth/restorative interface, favoring microleakage (14) and consequently decreasing the color stability of the resin composite, raising the susceptibility to staining.

The properties of composites are influenced not only by the characteristics of their fillers but also by the chemical structure of the matrix phase. Therefore, the greater stability of the P60 group may also be justified by the absence of the hydrophilic monomer TEGDMA 13 which has been replaced by a mixture of urethane dimethacrylate (UDMA), Bis-EMA and glycidyl methacrylate (Bis-GMA) which, according to the manufacturers, gives the composite greater hydrophobicity and are more resistant to color changes and less susceptible to water sorption because it has fewer hydrophilic aliphatic chains, presents less polarity, and contains carbamate linkages (10,15).

The Bulk-Fill, FF and SDR fluid resins tested in this study pigmented more significantly than the control group. This may be justified by a higher proportion of organic matrix compared to other resins, since water absorption occurs mainly from this matrix. The higher the content in organic matter, the less resistant the resin will be to hydrolytic degradation and water absorption and, consequently, the lower its color stability (10,11,15-17).

The color change observed can also be attributed to the silane agent, as the silanization of inorganic particles contributes to discoloration as a result of silane’s high propensity for water sorption (10,15,18). Increasing filler content in composite resins generally improves physical, chemical, and mechanical properties such as water absorption, color stability, and wear resistance (15,17).

Composite shade is an additional factor in resin staining because darker shades exhibit better color matching due to the presence of pigments. Possibly, universal shades undergo a greater degree of color change due to absence of pigments (17,19). In agreement with the obtained results, since the SDR group, which has a universal shade, was one of the groups that presented the highest color variation.

In addition to representing an indirect analysis of the degree of polymer conversion, color changes can cause considerable aesthetic damage, for example, when Bulk-Fill resins are inserted as a filling of proximal cavities with vestibular involvement. The Bulk-Fill restorative technique represents an important advance for dentistry, raising these resins to a level of restorative material with the most convenient and quick insertion technique in posterior teeth. Because it is a recently developed material, it still lacks long-term clinical studies that attest to its clinical performance in the most varied situations. The analysis of color variation showed better color stability for the Control Group compared to Bulk-Fill resins. Among Bulk-Fill resins, those with low viscosity showed the lowest color stability.
